# Factors Predicting Rubella Vaccination and Antibody in Pregnant Women in Japan: A Report from Pregnant Women Health Initiative

**DOI:** 10.3390/vaccines10050638

**Published:** 2022-04-19

**Authors:** Akiko Iwata, Kentaro Kurasawa, Kazumi Kubota, Mizuha Odagami, Shigeru Aoki, Mika Okuda, Etsuko Miyagi

**Affiliations:** 1 Department of Obstetrics and Gynecology, School of Medicine, Yokohama City University, Yokohama 236-0004, Japan; aiwata@yokohama-cu.ac.jp (A.I.); emiyagi@yokohama-cu.ac.jp (E.M.); 2 Department of Healthcare Information Management, School of Medicine, The University of Tokyo Hospital, Tokyo 113-8654, Japan; kkubota@m.u-tokyo.ac.jp; 3 Perinatal Center for Maternity and Neonates, Yokohama City University Medical Center, Yokohama 232-0024, Japan; mmmizuha@yahoo.co.jp (M.O.); smyyaoki@yokohama-cu.ac.jp (S.A.); 4 National Hospital Organization Yokohama Medical Center, Department of Obstetrics and Gynecology, Yokohama 245-8575, Japan; okuda.mika.ce@mail.hosp.go.jp

**Keywords:** rubella, rubella vaccine, rubella antibody, pregnant women, Japan, vaccination status, pregnant women health initiative

## Abstract

This study aimed to identify the factors predicting rubella vaccination status based on self-reported data and the presence of sufficient rubella antibody titers in pregnant women in Japan. We used the results of the nationwide questionnaire survey conducted at obstetric facilities in the Pregnant Women Health Initiative Project (PWHI), with 23 participating hospitals recruiting pregnant women from June 2018–November 2019. We extracted age, the number of deliveries, educational level, household income, pre-pregnancy smoking, and knowledge of rubella from questionnaires and medical records. We analyzed the association of rubella vaccination status and antibodies with each of these factors. We found that the number of previous deliveries, educational level, annual household income, smoking before pregnancy, and knowledge of rubella were factors predicting self-reported rubella vaccination status, while age and the number of previous deliveries were identified as factors predicting the presence of sufficient rubella antibody titers (32 folds or higher). Women considering pregnancy should be immunized against rubella to prevent congenital rubella syndrome in the future. Furthermore, social policies are needed to strongly encourage vaccination, especially for all citizens who were not given the opportunity or missed the chance to be vaccinated against rubella.

## 1. Introduction

**Figure 1:**
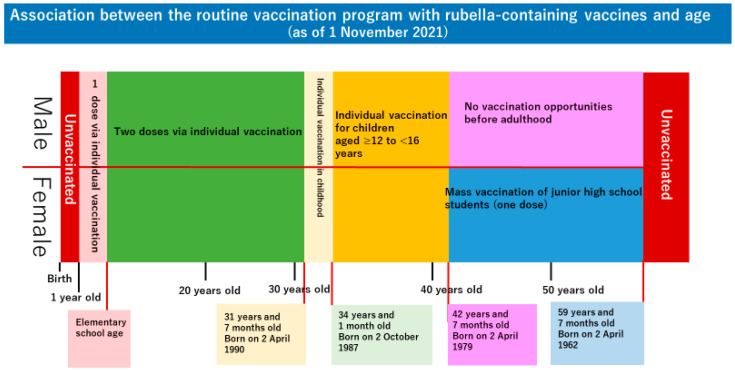
Association between the routine vaccination program with rubella-containing vaccines and age (as of 1 November 2021).

Rubella is a viral disease characterized by fever, rash, and lymphadenopathy. When a pregnant woman susceptible to rubella is infected with the rubella virus before approximately 20 weeks of pregnancy, her baby may develop congenital rubella syndrome (CRS) [1,2]. In Japan, a rubella outbreak occurred from 2012–2013; consequently, 45 babies were born with CRS [3,4,5,6,7]. Thus, the Ministry of Health, Labour, and Welfare issued the “Guidelines for the Prevention of Specific Infections: Rubella” to eliminate CRS prevalence in newborns as soon as possible and rubella by fiscal 2020 [8,9]. However, another rubella outbreak occurred from 2018–2019, and five infants were born with CRS [4,5]. Although the ministry aimed to constrain the prevalence of rubella before the Tokyo Olympic Paralympic Games, scheduled to be held in 2020, cases of CRS were registered in 2020 and 2021 [3]. The rubella outbreaks, repeating every few years, are associated with changes in the Japanese rubella vaccination program (Figure 1). In Japan, routine vaccination against rubella began in August 1977 [10]. With an emphasis on preventing infection in pregnant women, girls in junior high school were vaccinated en masse at school. In April 1995, the vaccination target was changed to infants of both sexes to prevent outbreaks of rubella in the society as a whole, and as a time-limited measure, vaccinations were administered to boys and girls in junior high school from April 1995 to September 2003. Furthermore, in June 2006, the previous one-dose vaccination regime was changed to a two-dose vaccination regime for one-year-old children (first period) and children in the year before entering elementary school (second period) [11]. Due to these program changes, men over 42 years of age currently had no opportunity to be vaccinated against rubella before adulthood, and some women of reproductive age have also missed vaccination opportunities [11,12]. These people became the hosts of the current rubella epidemic in Japan. Since 2020, the number of people infected with rubella has been decreasing [3]. This is presumably attributable to the restriction on human activities due to the coronavirus disease 2019 (COVID-19) pandemic. When human activities resume in the future, rubella outbreaks are expected to recur.

Pregnant women in Japan undergo screening for infectious diseases, including rubella, in the first trimester and receive necessary follow-up during pregnancy and after delivery based on screening results. To investigate the effects of this screening for infectious diseases on long-term maintenance and enhancement of maternal and child health, the Pregnant Women Health Initiative Project (PWHI) was launched. The PWHI is a study designed by Miyagi et al. and funded by the Health Labour Sciences Research Grant in 2018. This project conducts questionnaire surveys on hepatitis B, hepatitis C, rubella, human T-cell leukemia virus type 1 (HTLV-1), syphilis, and cervical cancer (human papillomavirus: HPV), for which pregnant women are screened in the first trimester. This study examines pregnant women for awareness and knowledge of rubella, vaccination, antibody seroprevalence, and socio-statistical or economic characteristics.

The following questions will allow effective implementation of preventive measures against rubella in Japan in the future: “Do pregnant women in Japan understand the extent of the rubella outbreak and take actions to protect their fetuses from CRS?” and “What characteristic makes pregnant women more likely to receive rubella vaccines and be seropositive for rubella antibodies?” We published the results of an interim analysis of data collected on rubella at a limited number of hospitals in Yokohama City [13]. In the present study, using the results of the nationwide questionnaire survey conducted at obstetric facilities in the PWHI, we investigated rubella vaccination and seroprevalence of rubella antibodies in pregnant women and aimed to identify factors predicting these features.

## 2. Materials and Methods

The PWHI was launched in April 2018, and 23 participating hospitals recruited pregnant women from June 2018–November 2019. Although these hospitals are dispersed across Japan, the most considerable number (11 hospitals) is located in Kanagawa Prefecture, where the PWHI was developed. Pregnant women (excluding those aged less than 20 years) scheduled for delivery at the participating hospitals received a written explanation of the objectives of the study and a leaflet containing the study participant number and QR (quick response) code from obstetricians at the participating hospitals. The women who wished to participate submitted the consent form with the study participant number to the obstetricians at the participating hospitals. Later, the participants sent an email to the study administration office at the address indicated by the QR code to complete the registration. After registration, the study administration office sent an email containing the link to the online questionnaire survey to the participants. At the survey site, they entered their study participant number and answered the questions. The questionnaire survey was conducted with SurveyMonkey^®^, a paid online survey tool with enhanced security. In the questionnaire survey, each participant was asked about the highest educational level, annual household income, lifestyle before pregnancy, physical conditions, results of screening for six infectious diseases (hepatitis B, hepatitis C, syphilis, rubella, HTLV-1, and HPV), vaccination status of the participant and her partner, and the knowledge of each infectious disease. The study collaborators provided data on the screening results of the participants, including age, the number of previous deliveries, and rubella antibody titers. The report rubella antibody titers (hemagglutination inhibition) were selected from the following options: “16 folds or lower” and “32 folds or higher”. From these data, those associated with rubella were extracted and analyzed. Regarding rubella vaccination status, the participants were asked, “Have you ever received rubella vaccination (including measles-rubella vaccines)?” and answered from the following options: “yes”, “no”, and “unknown”. To investigate factors predicting (self-reported) rubella vaccination status, participants who answered “unknown” were excluded, and the remaining participants were divided into the “yes” group and the “no” group. The characteristics of each group were compared. The following characteristics were extracted from the questionnaire survey responses and the data provided by the study collaborators: age, the number of previous deliveries, highest educational level, annual household income, smoking before pregnancy, rubella antibody titers, and knowledge of rubella (“Do you know about the outbreak of rubella in 2012–2013 in Japan?” and “Do you think rubella can affect your baby’s health directly?”). Subsequently, to investigate factors predicting the presence of sufficient rubella antibody titers, the same population was divided into two groups according to rubella antibody titers: the ≤16-fold group and the ≥32-fold group. The characteristics of each group were compared. In Japan, HI titers are widely used to evaluate rubella antibody titers. The most common international titer is the IgG international unit (IU/mL), and a titer of 10 IU/mL or higher is considered to be most protective against rubella [14]. The number of HI titers converted to IgG varies slightly across testing companies but is generally considered to be 15 IU/mL (12.340–18.476) [15]. The Clinical Guidelines for Obstetrical Practice in Japan recommend rubella vaccination for women who wish to conceive with HI titers ≤ 16 [16]. Therefore, in this study, we divided women into two groups: those with HI titers ≤ 16 and those with HI titers ≥ 32. In addition, rubella vaccination was recommended after delivery for pregnant women with HI titers ≤ 16.

### 2.1. Statistical Analysis

Binary logistic regression analysis was performed to examine whether there were differences in the rubella vaccine status based on socioeconomic and clinical characteristics of the participants (e.g., age, number of previous deliveries, or educational level) and whether those differences remained after adjusting for each background variable. The same analysis was performed for the rubella antibody status. Odds ratios (ORs) and corresponding 95% confidence intervals (CIs) were calculated. The level of significance was set to *p* < 0.05 (two-tailed). JMP Pro 15.0.0 (SAS Institute Japan, Tokyo, Japan) for Windows was used for statistical analysis.

### 2.2. Ethical Considerations

The protocol of this study was approved by each participating institution’s ethics review board at the Yokohama City University, aligning with the Ethical Guidelines for Medical and Health Research Involving Human Subjects.

## 3. Results

A total of 3003 pregnant women completed the questionnaire survey. Figure 1 shows the flowchart of the selection of study participants. Table 1 shows the study participants’ characteristics and responses to the questionnaire. The most common age group was in the 30s, and primiparous participants accounted for 47.0%. The proportion of pregnant women with a rubella antibody titer of 16 folds or lower was 29.8%. Those who reported having received rubella vaccines accounted for 68.1%, while those who reported that their partners had received them accounted for 48.6%.

**Figure 2:**
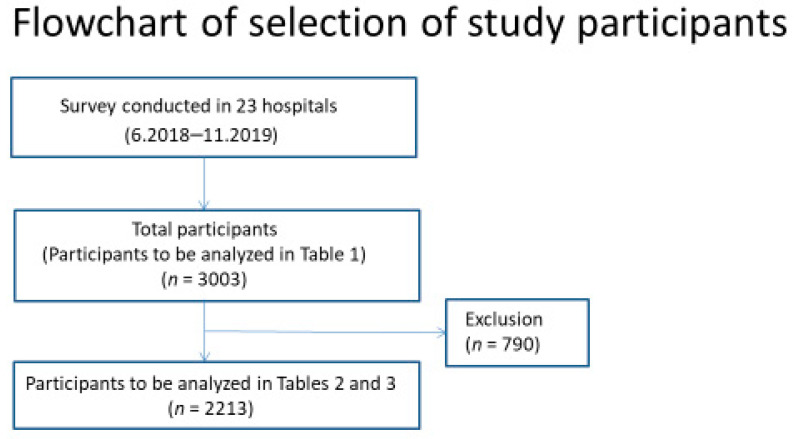
Flowchart of selection of study participants. Of the 3003 participants who completed the questionnaire survey, 790 did not answer questions regarding vaccination, educational level, annual household income, and so on or responded with “I do not know or want to answer.” After excluding these participants, 2213 participants were analyzed as shown in Table 2 and Table 3.

Table 2 shows the results of the comparison of characteristics between participants who reported to have received rubella vaccines and those who reported having been unvaccinated. Of the 3003 participants who completed the questionnaire survey, 790 answered a few questions regarding vaccination, educational level, annual household income, and so on by conveying that “they do not know or want to answer” or did not answer those questions. After these participants were excluded, 2213 participants were analyzed (Figure 2). Although younger participants were more likely to report being vaccinated, no significant difference was observed between age groups. Participants with fewer previous deliveries were more likely to report being vaccinated. The adjusted ORs were 0.53 (95% CI: 0.38–0.68, *p* < 0.0001) for participants with one previous delivery and 0.35 (95% CI: 0.24–0.50, *p* < 0.0001) for those with two or more previous deliveries, compared to those with no previous delivery. Participants with a higher educational level were more likely to report being vaccinated (adjusted OR = 1.89, *p* = 0.0003 for those with a junior college degree; adjusted OR = 2.02, *p* < 0.0001 for those with a university or higher degree compared to those with a high school diploma), and those with an annual household income of seven million yen or more were more likely to report being vaccinated than those with an annual household income of fewer than five million yen (adjusted OR = 1.42, *p* = 0.0366). Participants who had not smoked before pregnancy were more likely to report being vaccinated (adjusted OR = 1.90, *p* = 0.0002). Rubella antibody titers were not associated with vaccination status. Participants who correctly answered the questions regarding the knowledge of rubella (“Do you know about the outbreak of rubella in 2012–2013 in Japan?” and “Do you think rubella can affect your baby’s health directly?”) were more likely to report being vaccinated (adjusted OR = 1.63, *p* = 0.0002; adjusted OR = 1.33, *p* = 0.0458, respectively, for the questions).

Table 3 shows the results of the comparison of characteristics between the ≥32-fold and ≤16-fold groups according to rubella antibody titers. Participants in their 40s were more likely to have a rubella antibody titer of 32 folds or higher. The adjusted ORs were 0.60 for those in their 20s (*p* = 0.0048) and 0.55 for those in their 30s (*p* = 0.0003), compared to those in their 40s. Multiparous participants were more likely to have a rubella antibody titer of 32 folds or higher than primiparous participants. The adjusted ORs were 1.85 for those with one previous delivery (*p* < 0.0001) and 1.91 for those with two or more previous deliveries (*p* < 0.0001), compared to primiparous participants. No association was observed with the educational level, annual household income, smoking before pregnancy, self-reported rubella vaccination status, or knowledge of rubella.

## 4. Discussion

The questionnaire survey of pregnant women revealed two things. First, the factors predicting self-reported rubella vaccination status included the number of previous deliveries, educational level, annual household income, smoking before pregnancy, and the knowledge of rubella. Second, the factors predicting the presence of sufficient rubella antibody titers included age and the number of previous deliveries.

To protect pregnant women and their fetuses from rubella infection, vaccination before pregnancy is essential. We initially believed we could determine the rubella vaccination rate in pregnant women by asking about their vaccination status in a questionnaire survey. We wanted to identify factors predicting the vaccination status of pregnant women and use these factors to raise awareness of rubella vaccination. The results of this questionnaire survey showed that pregnant women with a higher educational level or a higher annual household income were more likely to report being vaccinated. Those who had not smoked before pregnancy and those who had knowledge of rubella were also more likely to report being vaccinated. Based on these results, we inferred that social background, lifestyle, and knowledge of the disease affected the vaccination status. If our assumption is correct, educational and raising awareness activities may effectively increase the vaccination rate. However, we could not explain why the vaccination rate was lower as the number of previous deliveries increased. Since postpartum vaccination is recommended for women with low rubella antibody titers in Japan, the vaccination rate should increase with more previous deliveries. However, the results contradicted this assumption. This may be because the results of this questionnaire survey might not accurately reflect the rubella vaccination rate. In this questionnaire survey, healthcare professionals were not required to check maternal and child health handbooks directly or ask pregnant women to submit their records of vaccination to determine their vaccination status. In Japan, vaccination certificates are not provided, making it difficult to know the vaccination history. Consequently, we examined “self-reported” rubella vaccination status and found that many participants (21.0%) answered that they did not know their vaccination status. While the rubella vaccination program has been changed in Japan, women in their 40s should have been vaccinated through mass vaccination at school when they were junior high school students [11]. However, this questionnaire survey showed that 39 of the 346 participants in their 40s (11.3%) reported not being vaccinated. We also excluded women in their 40s from our analysis because of the possibility of a larger recall bias. However, the results remained unchanged. This indicates that some participants completed the questionnaire survey without confirming they were vaccinated. The lack of association between self-reported rubella vaccination status and rubella antibody titers in this questionnaire survey also supports the possibility that self-reported rubella vaccination status is inaccurate. Trevisan et al. reported that self-reported vaccination history did not accurately predict immunity in five vaccine-preventable infectious diseases [17]. We consider that “self-reported” rubella vaccination status does not reflect actual rubella vaccination status but rather reflects the welfare of pregnant women in their own and their fetuses’ health. The reason why self-reported vaccination rates decrease as the number of deliveries increases is unknown. However, we suspect that women pay less attention to their bodies when they have more children and, as a result, may not check their vaccination history before pregnancy. Therefore, with a higher number of deliveries, pregnant women’s awareness of their vaccination history may become ambiguous, causing them to respond “not vaccinated” or “unknown” even when they are vaccinated.

The factors predicting the presence of sufficient rubella antibody titers included age and the number of previous deliveries. In Japan, the rubella vaccination program has been changed several times since the first introduction of rubella vaccines. As of 1 August 2018, the vaccination rate is high in women aged 39 years and 4 months or older (less than 56 years and 4 months) because they received a dose of the vaccine through mass vaccination when they were junior high school students. The vaccination rate in women aged 30 years and 10 months to 39 years and 4 months is considered low despite available opportunities for vaccination because the program was in transition. Women aged 28 years and 10 months to 30 years and 10 months had an opportunity to receive only one dose of the vaccine in their childhood. Women aged less than 28 years and 10 months had opportunities to receive two doses of the vaccine in their childhood [5]. Rubella antibody titers may reflect the vaccination status of these age groups. We consider that the results showed high antibody seroprevalence, especially in participants in their 40s, in whom the vaccination rate is reported to be high (although they had received only one dose). As described above, postpartum rubella vaccination is recommended for pregnant women with a rubella antibody titer of 16 folds or lower in Japan [16]. Women who have experienced pregnancy and delivery are expected to have their rubella antibody titers measured in screening during a previous pregnancy and to have been vaccinated during the postpartum period if their antibody titers are low. Thus, we consider that many pregnant women with at least one previous delivery had a rubella antibody titer of 32 folds or higher. Both age and the number of previous deliveries were expected to reflect the actual vaccination rate. Although antibody titers do not accurately indicate vaccination status, rubella antibody titers can be a factor in predicting vaccination status in a situation where the vaccination rate based on a questionnaire survey is less reliable. Unfortunately, the “undetectable rubella antibody titer (<8 folds)” and the “low antibody titer (8–16 folds)” were not distinguished for analyses in this questionnaire survey. To facilitate the entry of data by the study collaborators, the format was designed to group participants with an antibody titer of 16 folds or lower into one group. If we had analyzed antibody titers by dividing participants with an undetectable antibody titer and those with a low antibody titer, the antibody titers might have more accurately reflected rubella vaccination status.

The association between rubella antibodies in pregnant and prepregnant women and epidemiological factors has been reported in each country and region. Facciola et al. reported in a survey of pregnant women in Sicily, Italy, that rubella antibodies statistically correlate with age and educational levels [18]. Jonas et al. reported in a survey of women of reproductive age in Namibia that rubella antibody seroprevalence was higher in urban residents than in rural residents [19]. AlShamlan et al. reported in a survey of pregnant women in Saudi Arabia that rubella antibody seroprevalence decreases with increasing age [20]. Liu et al. reported in a survey of women preparing for pregnancy in rural China that women in regions with the low gross domestic product (GDP) per capita were more likely to be susceptible to rubella than women in regions with high GDP per capita [21]. Although factors affecting rubella antibodies vary among these reports, all reports concluded that susceptibility to rubella is affected by the environment where women live and their age. In Japan, no study has investigated rubella antibodies and multiple epidemiological factors in pregnant women. Regarding rubella antibody seronegativity in pregnant women by birth year, Hanaoka et al. and Okuda et al. reported that rubella antibody seronegativity is low in some age groups because the rubella vaccination program has been changed over time in Japan [22,23]. The results of the present study showed that rubella antibody titers in pregnant women in Japan were associated with age groups (probably affected by the changes in the rubella vaccination program) and the number of previous deliveries. Although our findings are partially consistent with those reported overseas, antibody titers did not differ among pregnant women with different educational levels or household incomes in Japan. Although the Japanese rubella vaccination program changes have caused confusion and a temporary decrease in the vaccination rate, the program can be regarded as having provided vaccines significantly to the target population.

In some people, rubella antibodies do not sufficiently increase or gradually decrease after rubella vaccination. In this questionnaire survey, 38% of primiparous women had a rubella antibody titer of 16 folds or lower. One of the future challenges of rubella preventive measures in Japan appears to be how to make women accurately understand their vaccination and immune status for rubella before their first pregnancy and how to vaccinate them before pregnancy if necessary. As reflected in the ‘self-reported’ vaccination status, it is possible that, in current Japan, the interest of pregnant women in their own and their fetuses’ health is affected by their social backgrounds and knowledge of the disease. Raising awareness and educational activities on rubella should be extensively implemented to encourage more women to practice preventive behaviors before pregnancy. Furthermore, it is important to eliminate rubella from women considering pregnancy and their families and from the entire society. The World Health Organization (WHO) currently recommends two vaccination doses for both boys and girls at an early age to eliminate rubella. By following this recommendation, some areas have successfully eliminated rubella [24,25]. Japan has also now adopted the program recommended by WHO. However, when rubella vaccination was first initiated, only adolescent girls were eligible. As a result, boys were not vaccinated. Furthermore, when the program was switched to vaccinating young boys and girls, vaccination coverage among women in the eligible years declined. These people became the hosts of the current rubella epidemic in Japan. Thus, a program was initiated in 2019 to provide opportunities to take a rubella antibody test and be vaccinated for men aged 41–58 years [11,26,27]. Some local governments subsidize rubella antibody tests and vaccines for women who are considering pregnancy [28,29,30]. A government policy promoting these programs and eliminating low rubella antibody prevalence could lead to the elimination of rubella from Japan.

The present study has some limitations. First, many surveyed items are based on self-reported questionnaire data. Specifically, the vaccination status was not verified by healthcare professionals, as described above. Second, the participating institutions are not evenly distributed regarding location. Although they are located across Japan, the most significant number is located in Kanagawa Prefecture, where the PWHI was developed. While all participating institutions are general hospitals that accept high-risk pregnant women, low-risk pregnant women in Japan often select small maternity hospitals. The vaccination rate and antibody seroprevalence may vary among regions and selected obstetric facilities. Third, the survey response rate could not be calculated. We surveyed 23 hospitals in Japan but do not know the number of people recruited overall. The study participant selection may be biased toward those who are health literate and have been vaccinated, thus affecting generalization. Next studies should have a way to collect information on the people approached and then participating to be able to calculate a response rate.

Based on the questionnaire survey of pregnant women, the present study identified the number of previous deliveries, educational level, annual household income, smoking before pregnancy, and knowledge of rubella as factors predicting self-reported rubella vaccination status, while age and the number of previous deliveries were identified as factors predicting the presence of sufficient rubella antibody titers. To immunize more women against rubella before pregnancy, the entire Japanese population needs to understand rubella, including women considering pregnancy. To this end, raising awareness and educational activities on rubella are essential.

## 5. Conclusions

The present study identified factors predicting rubella vaccination status based on self-reported data and the presence of sufficient rubella antibody titers in pregnant women in Japan. Although the prevalence of rubella decreased during 2020–2021 because of the COVID-19 pandemic, the Japanese rubella preventive measures are fragile with a possibility of a resurgence with increased human activities. The effect of herd immunity is also questionable. To prevent CRS in the future, women considering pregnancy should be immunized against rubella. Furthermore, social policies are necessary to strongly encourage vaccination, particularly for the citizens who had not received the opportunity or missed the chance to be vaccinated against rubella.

## Figures and Tables

**Table 1:** Study participants’ characteristics and responses to the questionnaire (*N* = 3003).

Characteristics and Responses to the Questionnaire	*n*	Rate, %
*Age group*		
20–29	707	23.9
30–39	1938	64.5
40–49	348	11.6
*Number of deliveries*		
0	1410	47.0
1	1141	38.0
2	368	12.3
≥3	84	2.8
*Educational level*		
≤High school graduate	573	19.1
Junior college graduate	1016	33.8
≥University graduate	1399	46.6
No answer	15	0.5
*Household income (ten thousand yen)*		
<500	921	30.7
≥500, <700	827	27.5
≥700	1088	36.2
No answer	167	5.6
*Did you smoke before pregnancy?*		
No	2590	86.2
Yes	408	13.6
No answer	5	0.2
*Rubella antibody titer (HI)*		
≥32	2102	70.2
≤16	893	29.8
*Do you know about the outbreak of rubella in 2012–2013 in Japan?*		
Yes	1900	63.3
No	1068	35.6
Unknown	35	1.2
*Do you think rubella can affect your baby’s health directly?*		
Yes	1082	36.0
No	1921	64.0
*Have you ever had the rubella vaccination?*		
Yes	2046	68.1
No	327	10.9
Unknown	630	21.0
*Has your partner ever had the rubella vaccination?*		
Yes	1459	48.6
No	335	11.2
Unknown	1209	40.3

**Table 2:** Factors predicting rubella vaccination among pregnant women (*N* = 2213).

	Have You Ever Had the Rubella Aaccination?		OR (95% CI)	*p* Value **	Adjusted * OR (95% CI)	*p* Value **
	Yes	No				
Age group						
20–29	460	66	1.20 (0.78–1.83)	0.4078	1.49 (0.96–2.34)	0.0773
30–39	1228	193	1.09 (0.75–1.59)	0.6390	1.28 (0.87–1.89)	0.2095
40–49	227	39	1.00		1.00	
Number of deliveries						
0	980	111	1.00		1.00	
1	687	124	0.63 (0.48–0.83)	0.0009	0.58 (0.38–0.68)	<0.0001
≥2	248	63	0.45 (0.32–0.63)	<0.0001	0.35 (0.24–0.50)	<0.0001
Educational level						
≤High school graduate	275	83	1.00		1.00	
Junior college graduate	653	98	2.01 (1.45–2.78)	<0.0001	1.89 (1.34–2.67)	0.0003
≥University graduate	987	117	2.55 (1.86–3.48)	<0.0001	2.02 (1.42–2.88)	<0.0001
Household income (ten thousand yen)						
<500	549	113	1.00		1.00	
≥500, <700	574	88	1.34 (0.99–1.81)	0.0561	1.28 (0.93–1.76)	0.1302
≥700	792	97	1.68 (1.25–2.25)	0.0005	1.42 (1.02–1.96)	0.0366
Did you smoke before pregnancy?						
No	1717	236	2.28 (1.66–3.12)	<0.0001	1.90 (1.35–2.67)	0.0002
Yes	198	62	1.00		1.00	
Rubella antibody titer (HI Titer)						
≥32×	1324	198	1.13 (0.87–1.47)	0.3505	1.15 (0.88–1.51)	0.3100
≤16×	591	100	1.00		1.00	
Do you know about the outbreak of rubella in 2012–2013 in Japan?						
Yes	1327	171	1.68 (1.31–2.15)	<0.0001	1.63 (1.26–2.11)	0.0002
No	588	127	1.00		1.00	
Do you think rubella can affect your baby’s health directly?						
Yes	1151	210	1.58 (1.22–2.06)	0.0007	1.33 (1.01–1.75)	0.0458
No	764	88	1.00		1.00	

* Model includes all variables for which values are shown in the column. ** The red colored numbers indicate *p* < 0.05.

**Table 3:** Factors predicting rubella antibody titer among pregnant women (*N* = 2213).

	Rubella Antibody Titer (HI Titer)		OR (95% CI)	*p* Value **	Adjusted * OR (95% CI)	*p* Value **
	≥32×	≤16×				
Age group						
20–29	351	175	0.55 (0.39–0.77)	0.0006	0.60 (0.42–0.86)	0.0048
30–39	962	459	0.57 (0.42–0.78)	0.0005	0.55 (0.40–0.76)	0.0003
40–49	209	57	1.00		1.00	
Number of deliveries						
0	676	415	1.00		1.00	
1	611	200	1.88 (1.53–2.29)	<0.0001	1.85 (1.50–2.27)	<0.0001
≥2	235	76	1.90 (1.43–2.53)	<0.0001	1.91 (1.42–2.56)	<0.0001
Educational level						
≤High school graduate	222	136	1.00		1.00	
Junior college graduate	516	235	1.35 (1.03–1.75)	0.0273	1.13 (0.86–1.49)	0.3743
≥University graduate	784	320	1.50 (1.17–1.03)	0.0014	1.23 (0.93–1.63)	0.1402
Household income (ten thousand yen)						
<500	426	236	1.00		1.00	
≥500, <700	454	208	1.21 (0.96–1.52)	0.1033	1.03 (0.81–1.31)	0.8144
≥700	642	247	1.44 (1.16–1.79)	0.0010	1.13 (0.89–1.43)	0.3261
Did you smoke before pregnancy?						
No	1354	599	1.24 (0.94–1.62)	0.1239	1.04 (0.78–1.39)	0.7771
Yes	168	92	1.00			
Have you ever had the rubella vaccination?						
Yes	1324	591	1.13 (0.87–1.47)	0.3505	1.16 (0.88–1.52)	0.2889
No	198	100	1.00		1.00	
Do you know about the outbreak of rubella in 2012–2013 in Japan?						
Yes	1054	444	1.25 (1.04–1.51)	0.0200	1.13 (0.93–1.37)	0.2335
No	468	247	1.00		1.00	
Do you think rubella can affect your baby’s health directly?						
Yes	610	242	1.24 (1.03–1.50)	0.0236	1.17 (0.96–1.42)	0.1218
No	912	449	1.00		1.00	

* Model includes all variables for which values are shown in the column. ** The red colored numbers indicate *p* < 0.05.

## Data Availability

The data that support the findings of this study are available from the corresponding author, K.K., upon reasonable request.
